# Brachial plexus magnetic resonance imaging differentiates between inflammatory neuropathies and does not predict disease course

**DOI:** 10.1002/brb3.632

**Published:** 2017-04-04

**Authors:** Bas A. Jongbloed, Jeroen W. Bos, Dirk Rutgers, Willem Ludo van der Pol, Leonard H. van den Berg

**Affiliations:** ^1^ Department of NeurologyUniversity Medical Center UtrechtUtrechtNetherlands; ^2^ Department of RadiologyUniversity Medical Center UtrechtUtrechtNetherlands

**Keywords:** Brachial plexus MRI, inflammatory neuropathies, multifocal motor neuropathy, Lewis‐Sumner syndrome, chronic inflammatory demyelinating polyradiculoneuropathy

## Abstract

**Objective:**

The main objective of this study was to evaluate the correlation between the distribution of brachial plexus magnetic resonance imaging (MRI) abnormalities and clinical weakness, and to evaluate the value of brachial plexus MRI in predicting disease course and response to treatment in multifocal motor neuropathy (MMN), Lewis‐Sumner syndrome (LSS) and chronic inflammatory demyelinating polyradiculoneuropathy (CIDP).

**Methods:**

Sixty‐seven patients with an inflammatory neuropathy diagnosed at our tertiary referral center for neuromuscular diseases had undergone bilateral T2‐weighted short tau inversion recovery (STIR) MRI of the brachial plexus. We obtained clinical follow‐up data and scored all MRIs for abnormalities and the symmetry of their distribution.

**Results:**

Brachial plexus MRI abnormalities were detected in 45% of the patients. An abnormal MRI did not predict disease course in terms of patterns of weakness, sensory disturbances or response to treatment. Within the spectrum of radiological abnormalities, asymmetrical clinical syndromes, MMN and LSS were significantly associated with asymmetrical radiological abnormalities, whereas symmetrical abnormalities predominated in CIDP (*p *< .001, phi 0.791).

**Conclusion:**

T2 STIR brachial plexus MRI abnormalities correspond with the distribution of neurological deficits in inflammatory neuropathies, but do not correlate with specific clinical characteristics, response to treatment or disease course.

## 1 Introduction

Hyperintensity and hypertrophy of cervical nerves and nerve roots using T2‐weighted short tau inversion recovery (STIR) magnetic resonance imaging (MRI) techniques have been described in patients with various types of demyelinating inflammatory neuropathies, including multifocal motor neuropathy (MMN) (Van Es et al., 1997), chronic inflammatory demyelinating polyradiculoneuropathy (CIDP) (Adachi et al., 2011; Sinclair et al., 2011; Van Es et al., 1997), Lewis‐Sumner syndrome (LSS) (Rajabally, Knopp, Martin‐Lamb, & Morlese, 2014; Van den Berg‐Vos et al., 2000), and polyneuropathy associated with IgM monoclonal gammopathy (Eurelings, Notermans, Van de Donk, & Lokhorst, 2001). Abnormalities on MR imaging of the brachial plexus are an argument for inflammatory neuropathies, particularly when nerve conduction studies are not fully conclusive. Therefore, positive findings on brachial plexus MRI have been included in the diagnostic consensus criteria for CIDP and MMN (Joint Task Force of the EFNS and the PNS for CIDP, 2010; Joint Task Force of the EFNS and the PNS for MMN, 2010).

It is unclear whether patterns of brachial plexus MRI abnormalities are associated with specific inflammatory neuropathies. This is important as different neuropathies may require specific therapeutic strategies (Nobile‐Orazio, 2014; Pestronk et al., 1988; van Schaik, van den Berg, de Haan, & Vermeulen, 2005). Comparative studies of brachial plexus MRI have not been performed. A number of smaller studies have mainly suggested that asymmetry is associated with MMN or LSS rather than CIDP, but others could not confirm this (Rajabally et al., 2014; Van den Berg‐Vos et al., 2000; Van Es et al., 1997). Moreover, it is not known whether MRI abnormalities predict disease course or response to treatment.

We therefore investigated patterns of brachial plexus MRI abnormalities in patients within the spectrum of inflammatory neuropathies, together with clinical follow‐up data of all patients who had undergone an MRI to study their disease course and response to treatment.

## 2 Patients and Methods

### 2.1 Patients

All patients who were diagnosed with MMN, LSS or CIDP at the tertiary referral neuromuscular outpatient clinic of the University Medical Center Utrecht between 1996‐2015 were screened and those who had undergone bilateral T2 STIR MR imaging of the brachial plexus were included in this study (Van Es, 2001). We checked the diagnosis of MMN and CIDP, including the Lewis‐Sumner variant as described in the most recent diagnostic consensus criteria for MMN and CIDP (Joint Task Force of the EFNS and the PNS for CIDP, 2010; Joint Task Force of the EFNS and the PNS for MMN, 2010).

### 2.2 Clinical examination

Description of the neurological examination at the time of the MRI and at the last follow‐up as reported in patient files was used to assess (a)symmetry in muscle strength and sensory disturbances. Asymmetry was defined as a minimum difference of two Medical research Council (MRC) points of tested muscle groups per limb. Sensory deficits were scored as being present or absent for each individual limb. Clinical response to treatment was defined as clinical improvement reported by the patient, or improving in muscle strength by gaining at least 1 MRC point in a muscle group (Léger et al., 2008).

### 2.3 Brachial plexus MRI

MRIs of the brachial plexus were made between 1999 and 2015 using a previously described protocol, including bilateral T2 STIR imaging in the coronalplane on a 0.5‐1.5T MRI scan, with slice thickness of 3.0 mm, a b‐value of 0 and echo time of 60‐100 ms (Van Es et al., 1997). All MRIs were made before and after the administration of gadolinium. Bilateral coronal T2 STIR images of the brachial plexus were used to identify abnormalities, that is signal hyperintensity and cervical nerve (root) thickening. All available MRIs were scored by an experienced neuroradiologist and all MRIs were re‐examined by two of the authors (BJ and JB). In case of doubt, an experienced neuroradiologist (DR), blinded for the diagnosis, was consulted. All abnormal MRIs were scored as symmetrical or asymmetrical by visual interpretation of nerve intensities and nerve thickness.

The locally appointed ethics committee of the UMC Utrecht approved this study and they gave dispensation for informed consent from the included subjects as it is a retrospective medical file investigation.

### 2.4 Statistical analysis

Statistical analyses were carried out using IBM SPSS 22.0 software. For continuous data, between‐group comparisons were made using Kruskal‐Wallis H testing with post hoc Mann‐Whitney U or Student's t‐testing, as appropriate. Bonferroni correction for multiple testing was applied. Categorical variables were analyzed using a chi‐square or Fisher's exact test. The phi coefficient was used as a measure of association between binary variables. A *p*‐value <.05 was considered statistically significant.

## 3 Results

### 3.1 Patients

We identified a total of 267 patients with an inflammatory neuropathy, of which brachial plexus MRI had been performed in 67 patients; 40 patients had MMN, nine patients had LSS and 18 patients had CIDP. Of the CIDP patients, six had a pure‐motor variant. Patient characteristics are listed in Table 1.

**Table 1:** Patient characteristics

	MMN	LSS	CIDP
Number of patients	40	9	18
Male gender	29 (73%)	9 (100%)	8 (44%)
Age at diagnosis^a^^,^ b	48 (26–73)	52 (20–71)	52 (30–62)
Time to diagnosis^b^^,^ c	43 (3–158)	83 (15–240)	11 (5–132)
Diagnosis (%)			
Definite	26 (65)	–	14 (78)
Probable	8 (20)	–	3 (17)
Possible	6 (15)	–	1 (5)
Site of onset (%)			
Arm(s)	33 (82.5)	7 (78)	2 (11)
Leg(s)	5 (12.5)	1 (11)	10 (56)
Arm(s) and leg(s)	2 (5)	1 (11)	6 (33)

MMN, Multifocal Motor Neuropathy; LSS, Lewis‐Sumner Syndrome; CIDP, Chronic Inflammatory Demyelinating Polyneuropathy.

Baseline characteristics of MMN, LSS, and CIDP patients.

a Median age in years (range).

b Mann‐Whitney U‐test *p* < .05.

c Median time in months (range).

Multifocal motor neuropathy patients were significantly younger than CIDP patients at the time of diagnosis (*p *< .001). Time to diagnosis was significantly longer in MMN and LSS than for CIDP (*p *< .001).

### 3.2 Brachial plexus MRI

**Figure 1:**
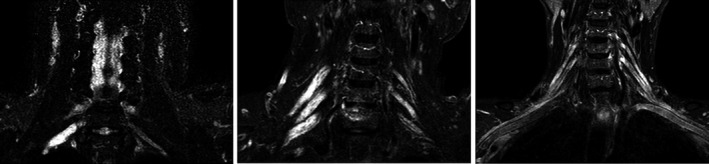
Hypertrophy and nerve thickening on bilateral coronal T2 STIR MR imaging of the brachial plexus in inflammatory neuropathies. Left: unilateral hypertrophy and thickening of cervical nerve roots in an multifocal motor neuropathy (MMN) patient. Middle: Bilateral and asymmetrical abnormalities in an Lewis‐Sumner syndrome (LSS) patient. Right: Bilateral and symmetrical abnormalities in a chronic inflammatory demyelinating polyradiculoneuropathy (CIDP) patient

MRI abnormalities were detected in 30/67 (45%) patients (Table 2, Figure 1). Abnormalities were asymmetrical in 12 of 14 (86%) patients with MMN and in all 5 (100%) patients with LSS. In contrast, only 9% of CIDP patients showed asymmetrical abnormalities. Unilateral abnormalities were found only in MMN and LSS. No contrast enhancement was found in any of the disease groups.

**Table 2:** Brachial plexus MRI characteristics

	MMN	LSS	CIDP
Patients (N)	40	9	18
Abnormal MRI (%)	14 (35)	5 (56)	11 (61)
Thickening	12 (86)	5 (100)	10 (91)
Hyperintensity	12 (86)	4 (80)	8 (73)
Bilateral abnormalities	9 (64)	2 (40)	11 (100)
Asymmetry	12 (86)	0	1 (9)

MMN, Multifocal Motor Neuropathy; LSS, Lewis‐Sumner Syndrome; CIDP, Chronic Inflammatory Demyelinating Polyneuropathy.

Characteristics of brachial plexus MRIs in MMN, LSS and CIDP patients.

Evaluation of the correlation between clinical and radiological asymmetry revealed that asymmetric MRI abnormalities were associated with asymmetric weakness in 12 of 14 patients with MMN (86%) and in all five patients with LSS (100%) (Table 3). Similarly, symmetry of abnormalities on MRI and on clinical examination coincided (9/11 (82%)) in CIDP patients.

**Table 3:** Clinical symmetry versus symmetry of abnormalities of brachial plexus MRI in inflammatory neuropathies

	MMN *N *= 14 (%)	LSS *N *= 5 (%)	CIDP *N *= 11 (%)
*MRI abnormalities*			
Symmetrical			
Clinical findings			
Symmetrical	1 (7)	0	9 (82)
Asymmetrical	1 (7)	0	1 (9)
*Asymmetrical*			
Clinical findings			
Symmetrical	0	0	0
Asymmetrical	12 (86)	5 (100)	1 (9)

MMN, Multifocal Motor Neuropathy; LSS, Lewis‐Sumner Syndrome; CIDP, Chronic Inflammatory Demyelinating Polyneuropathy.

Correlation between symmetry of abnormalities found on MR imaging of the brachial plexus and symmetry of clinical findings at the time of the MRI in MMN, LSS and CIDP patients.

### 3.3 Correlation clinical disease course versus MRI

No significant differences in clinical presentation, response to treatment and follow‐up data were detected between patients with and without abnormalities on MRI per diagnosis (Table 4).

**Table 4:** Patient characteristics and disease course in patients with and without brachial plexus abnormalities

Brachial plexus MRI	MMN		LSS		CIDP	
	Normal (*n *= 26)	Abnormal (*n *= 14)	Normal (*n *= 4)	Abnormal (*n *= 5)	Normal (*n *= 7)	Abnormal (*n *= 11)
Male gender	21 (81%)	8 (57%)	4 (100%)	4 (80%)	2	6
Age at diagnosis^a^	48 (31–65)	43 (26–60)	63 (44–70)	46 (20–71)		
Symptom distribution^b^	20/4/2	13/1/0	4/0/0	2/1/1	0/3/3	2/6/2
Anti‐GM1 IgM positive	6/12	4/5	‐	‐	0/1	0/1
Improvement after IVIg	24/26	13/14	4/4	2/2	4/4	6/7
Follow‐up^c^	48 (3–210)		53 (16–97)		65 (1–318)	
Sensory disturbances	0/26	0/14	4/4	4/4	5/7	7/10
Proximal leg weakness	2/26	2/14	0/4	0/4	3/7	9/10
Symmetrical weakness	0/26	2/14	0/4	0/4	6/6	8/10

MMN, Multifocal Motor Neuropathy; LSS, Lewis‐Sumner Syndrome; CIDP, Chronic Inflammatory Demyelinating Polyneuropathy.

Clinical information at onset and at the last follow‐up moment in inflammatory neuropathies. Within each inflammatory neuropathy, no significant difference was found per clinical item between patients with normal and abnormal MRIs of the brachial plexus.

a Median age in years (range),

b Distribution shown at onset in arm(s)/leg(s)/both,

c Median follow‐up in months (range) of 40 MMN, 8 LSS and 17 CIDP patients.

Follow‐up studies of all 67 patients who underwent an MRI revealed that the diagnosis was altered only twice, namely from MMN into CIDP.

### 3.4 Pooled data results

Pooled data analysis (Table 5) showed that clinical and brachial plexus MRI asymmetry concurred in the majority of MMN and LSS cases (17 of 19 (90%)). MRI abnormalities were symmetrical in 10 of 11 patients with CIDP (91%). Combining the data from our study showed that clinical and radiological asymmetry coincided (*p* < .001, phi 0.791).

**Table 5:** Pooled data of symmetry of abnormalities on MRI versus symmetry of neuropathies

	MMN & LSS	CIDP	Total
MRI abnormalities			
Symmetrical	2	10	12
Asymmetrical	17	1	18
Total	19	11	30

MMN, Multifocal Motor Neuropathy; LSS, Lewis‐Sumner Syndrome; CIDP, Chronic Inflammatory Demyelinating Polyneuropathy.

Pooled data of asymmetrical neuropathies (MMN and LSS) and symmetrical neuropathies (CIDP), and symmetry of abnormalities on MR imaging of the brachial plexus.

## 4 Discussion

This study shows that (a)symmetry of abnormalities on brachial plexus MR imaging is associated with the distribution of clinical findings in patients with an inflammatory neuropathy. Hence, symmetrical distribution of MRI abnormalities is strongly associated with CIDP, whereas asymmetry suggests a diagnosis of MMN or LSS. Asymmetry of brachial plexus MRI abnormalities is associated with persisting asymmetry of weakness at follow‐up indicating that radiological asymmetry is not a snapshot in time in the evolution to a symmetric disease, that is CIDP. Moreover, presence of (patterns of) MRI abnormalities do not predict a beneficial response to treatment with immunoglobulins.

Brachial plexus MRI is an established technique in the diagnostic work‐up of patients suspected of an inflammatory neuropathy, but its predictive value for disease course or the relevance of specific patterns of weakness have not been studied. Moreover, comparative studies combining MR data of MMN, LSS and CIDP patients have not been performed. Symmetric brachial plexus abnormalities have been described using high‐resolution ultrasound (HRUS) in asymmetric inflammatory neuropathies (Beekman et al., 2005). This discrepancy in results between HRUS and MRI may be caused by the lack of standardized MRI or HRUS criteria defining abnormality and asymmetry, but may also reflect differences in diagnostic sensitivity and specificity.

In two patients with an initial diagnosis of MMN, the diagnosis was changed into CIDP during follow‐up. One patient had both symmetric weakness and MRI abnormalities early in the disease course. He developed proximal leg weakness and his cerebrospinal fluid revealed an elevated protein level (>1 g/L), which were considered incompatible with MMN. The other patient had asymmetric weakness and MRI abnormalities at onset, but this developed rapidly into symmetrical weakness with prominent proximal leg weakness, and he showed remission of symptoms after plasmapheresis and corticosteroids; these findings are all compatible with a diagnosis of CIDP.

As a result of the rarity of inflammatory neuropathies, a drawback of our study are the relatively low number of inclusions and its retrospective nature, possibly giving rise to a selected study population. Indeed, the number of patients diagnosed with probable and possible MMN compared with definite MMN were higher in this study than in the Dutch population‐based MMN cohort, (Cats et al., 2010) indicating that at least part of the brachial plexus MRIs were made in the diagnostic work‐up of patients suspected of having MMN. Yet, we believe this increases the clinical relevance of the correlation between radiological and clinical findings we found in this study. Although we report the largest study on MR imaging of the brachial plexus of MMN, LSS, and CIDP patients so far, the total number of included patients may be relatively small and results should be interpreted with care. For MR imaging of the brachial plexus there is no clear definition of symmetry of nerve enlargement or hyperintensity and as such, the interrater reliability may be limited. However, the authors agreed with 90% of the reports of the MRIs. In the remaining 10% of which there was disagreement, MRIs were re‐examined by an experienced neuroradiologist (DR), blinded to the diagnosis.

This study shows that patients with a demyelinating inflammatory neuropathy and an abnormal MRI of the brachial plexus do not represent a subgroup of patients with a specific clinical presentation, disease course or response to immunoglobulin treatment as compared to patients with a normal brachial plexus MRI. The correlation between radiological and clinical abnormalities needs further validation, ideally in cohorts of consecutive patients in whom brachial plexus MRIs are made as an ancillary tool in the work‐up of inflammatory neuropathies. Recognition of asymmetry may help to differentiate MMN or LSS from CIDP and therefore may contribute to the selection of appropriate treatment strategies.

## Author Contributions

B.A. Jongbloed: Acquisition of data, contribution to study design, statistical analysis and interpretation of data, drafting/revision of the manuscript. J.W. Bos: Acquisition of data, statistical analysis and interpretation of data, drafting/revision of the manuscript. D. Rutgers: Interpretation of the MRIs, drafting/revision of the manuscript. W.L. van der Pol and L.H. van den Berg: Interpretation of data, drafting/revision of the manuscript.

## Disclosures

B.A. Jongbloed reports no disclosures. J.W. Bos reports no disclosures. D. Rutgers reports no disclosures. L.H. van den Berg serves on scientific advisory boards for the Prinses Beatrix Spierfonds, Thierry Latran Foundation, Biogen Idec and Cytokinetics; received an educational grant from Baxalta; serves on the editorial board of Amyotrophic Lateral Sclerosis And Frontotemporal Degeration and the Journal of Neurology, Neurosurgery and Psychiatry; and receives research support from the Prinses Beatrix Spierfonds, Netherlands ALS Foundation, The European Community's Health Seventh Framework Programme (grant agreement n° 259867), The Netherlands Organization for Health Research and Development (Vici Scheme, JPND (SOPHIA, STRENGTH, ALSCare)). W.L. van der Pol receives research support from the Prinses Beatrix Spierfonds; The Netherlands ALS Foundation; Stichting Spieren voor Spieren.
